# Karyotype Characterization of *In Vivo*- and *In Vitro*-Derived Porcine Parthenogenetic Cell Lines

**DOI:** 10.1371/journal.pone.0097974

**Published:** 2014-05-20

**Authors:** Qiang Liu, Manling Zhang, Dongxia Hou, Xuejie Han, Yong Jin, Lihua Zhao, Xiaowei Nie, Xin Zhou, Ting Yun, Yuhang Zhao, Xianghua Huang, Daorong Hou, Ning Yang, Zhaoqiang Wu, Xueling Li, Rongfeng Li

**Affiliations:** 1 State Key Laboratory of Reproductive Medicine, Nanjing Medical University, Nanjing, Jiangsu, China; 2 Jiangsu Key Laboratory of Xenotransplantation, Nanjing Medical University, Nanjing, Jiangsu, China; 3 The Key Laboratory of the National Education Ministry for Mammalian Reproductive Biology and Biotechnology, Inner Mongolia University, Hohhot, Inner Mongolia, China; Institute of Zoology, Chinese Academy of Sciences, China

## Abstract

Mammalian haploid cell lines provide useful tools for both genetic studies and transgenic animal production. To derive porcine haploid cells, three sets of experiments were conducted. First, genomes of blastomeres from 8-cell to 16-cell porcine parthenogenetically activated (PA) embryos were examined by chromosome spread analysis. An intact haploid genome was maintained by 48.15% of blastomeres. Based on this result, two major approaches for amplifying the haploid cell population were tested. First, embryonic stem-like (ES-like) cells were cultured from PA blastocyst stage embryos, and second, fetal fibroblasts from implanted day 30 PA fetuses were cultured. A total of six ES-like cell lines were derived from PA blastocysts. No chromosome spread with exactly 19 chromosomes (the normal haploid complement) was found. Four cell lines showed a tendency to develop to polyploidy (more than 38 chromosomes). The karyotypes of the fetal fibroblasts showed different abnormalities. Cells with 19–38 chromosomes were the predominant karyotype (59.48–60.91%). The diploid cells were the second most observed karyotype (16.17%–22.73%). Although a low percentage (3.45–8.33%) of cells with 19 chromosomes were detected in 18.52% of the fetus-derived cell lines, these cells were not authentic haploid cells since they exhibited random losses or gains of some chromosomes. The haploid fibroblasts were not efficiently enriched via flow cytometry sorting. On the contrary, the diploid cells were efficiently enriched. The enriched parthenogenetic diploid cells showed normal karyotypes and expressed paternally imprinted genes at extremely low levels. We concluded that only a limited number of authentic haploid cells could be obtained from porcine cleavage-stage parthenogenetic embryos. Unlike mouse, the karyotype of porcine PA embryo-derived haploid cells is not stable, long-term culture of parthenogenetic embryos, either *in vivo* or *in vitro*, resulted in abnormal karyotypes. The porcine PA embryo-derived diploid fibroblasts enriched from sorting might be candidate cells for paternally imprinted gene research.

## Introduction

Most animal cells exist in the diploid condition, possessing two homologues of each chromosome. By contrast, haploid cells have one set of chromosomes. Haploid cell lines have advantages for forward and reverse genetic screens by avoiding allelic effects and thus exhibit an obvious phenotype in genetic modified individuals, especially when investigating polygenic traits [1]. The haploid cell is also an effective tool to obtain live genetically modified mammals through transfer of a genetically modified androgenetic haploid cell into oocyte or transfer of both parthenogenetic haploid cell and sperm into enucleated oocyte [2], [3]. Haploid cells may also be useful to preserve genetic material of female mammals with a desired phenotype.

In mammals, haploid cells are usually restricted to sperm and oocyte which are cells derived from primordial germ cells and that are unable to proliferate [4]. The derivation of haploid cell lines has been achieved in fish embryonic stem cells [5] and in human KBM-7 leukemia cells [6]. Although haploid cells were found in mouse embryos at the morula or blastocyst stages more than 30 years ago [1], [7], [8], these cells were either not examined for their genetic integrity or were shown to possess a near haploid chromosome spread due to the absence of some chromosomes [8]. Recently, mammalian haploid cells exhibiting genetic stability were successfully obtained from mouse [9]–[11], rat [12], and non-human primate [13] cells.

Parthenogenetic activation of oocytes combined with cell culture techniques has been used to derive haploid cells. Mouse oocytes activated without disruption of the secondary polar body extrusion can develop to the blastocyst stage with an intact haploid chromosome complement [8]. By injecting sperm into enucleated MII phase oocytes, researchers have derived haploid androgenetic embryos [9], [14]. These parthenogenetic and androgenetic embryos could be further cultured to obtain an embryonic stem-like (ES-like) cell population with a high percentage (60–80%) of haploid stem cells after sorting every three to four passages [11]. These haploid stem cells were authentic haploid cells, as shown via chromosome spread, comparative genomic hybridization (CGH), or deep sequencing. To our knowledge, with the exception of mouse, rat and monkey, no other mammalian haploid cell lines have been reported to date.

Pigs provide an excellent model for human disease due to the similarities to humans in anatomy and function. Pig haploid cells will be very useful for basic research and transgenic pig production. Two groups have studied haploid blastomeres at the morula stage of porcine PA embryos [15], [16], but they did not identify the genome integrity of the cells. Therefore, whether authentic haploid blastomeres exist within porcine PA embryos has not been validated.

Except for the use of haploid cells, the PA embryo-derived diploid cells, due to the absence of the paternal genetic material, could provide a model system to discover unknown paternally imprinted genes through comparisons to normal fibroblasts. Nikaido et al. used Large-Scale expression profiling of parthenogenote and androgenote mouse embryos to discover imprinted transcripts [17]. Sritanaudomchai et al. discovered a novel imprinted gene by transcriptional analysis of parthenogenetic rhesus monkey embryonic stem cells [18]. Currently, only 30 imprinted genes have been identified in the porcine genome due to the lack of materials and the lack of information on SNPs (www.geneimprint.com/site/genes-by-species.Sus+scrofa).

In this study, we used porcine oocytes to produce a high percentage of haploid PA embryos. We first confirmed that authentic haploid cells were present in the cleavage-stage PA embryos. Based on this confirmation, parthenogenetic embryos were used for ES-like cell culture or embryo transfer, followed by fetus collection, fibroblast culture and fibroblast sorting to obtain large populations of cells with a high percentage of genetic intact haploid cells. The possibility that porcine PA embryo-derived diploid cells could be used for paternally imprinted gene research was also investigated.

## Materials and Methods

### General Experimental Design

Porcine oocytes were activated without cytochalasin B to avoid generation of diploid embryos. Three major sets of experiments were performed with these presumed haploid PA embryos to obtain large amounts of haploid porcine cells.

The first set of experiments was used to confirm that there are genetically intact haploid blastomeres in porcine haploid PA embryos at early cleavage stages. To evaluate the chromosome spread, the 8–16 cell stages of PA embryos were dissected and a chromosome spread analysis was performed. To further evaluate each of the chromosomes and verify the genetic integrity of the haploid cells, we performed a G-banding chromosome analysis.

Because the availability of these haploid blastomeres is severely limited, we conducted additional experiments to test whether we could derive haploid stem-like cells from the PA embryos using traditional stem-cell culture methods. Both inner cell masses (ICMs) with the trophectoderm cells stripped and entire PA blastocysts with the zona pellucida removed were cultured in porcine stem-cell medium with Fibroblast Growth Factor (FGF). Chromosome spread analysis of the derived stem-like cells was performed and no haploid cells were observed. Cells derived via this culture method showed a tendency to develop aneuploidy or polyploidy.

Lastly, porcine PA embryos were transferred to recipient gilts on the second day after parthenogenetic activation to determine if genetically intact haploid cells could be recovered from fetuses. The fetuses were collected on day 30 after embryo transfer, fetal fibroblast cell lines were established and fibroblast chromosome spread analysis was conducted. A small fraction of fibroblasts were found to have 19 chromosomes in some fetal fibroblast cell lines. The cell lines with putative haploid cells were sorted using flow cytometry to enrich haploid fibroblasts. Chromosome spreads of the concentrated haploid cells were conducted to determine the enrichment efficiency. The genetic integrity of the fetal fibroblasts was investigated via G-banding analysis.

In addition, the pig PA fetuses were fixed for tissue sectioning and HE staining. Transcriptional profiling of diploid PA fetal fibroblasts was conducted via microarray analysis followed by qPCR confirmation. These experiments enable us to understand the abnormal development of the porcine PA fetus or find candidate genes related to abnormal development. Diploid PA fetal fibroblasts may be candidate cells for discovery of unknown paternally imprinted genes.

### Animal Care and Use

All experiments with animals were conducted in accordance with the Guide for the Care and Use of Laboratory Research Animals and were approved by the Animal Care and Use Committees of Nanjing Medical University and Inner Mongolia University.

### Chemicals

All chemicals were purchased from Sigma (St. Louis, MO) unless otherwise indicated.

### Oocyte Collection and *In vitro* Maturation

Porcine ovaries were collected from local slaughterhouses, the Xikouzi Slaughterhouse in Hohhot and the Slaughterhouse of Meat Processing Factory in Nanjing, and incubated in 0.9% NaCl at 37°C until use. The cumulus-oocyte complexes (COCs) and follicular fluid were aspirated using an 18 gauge disposable needle from mature follicles (3–6 mm in diameter) and transferred into a 15 ml conical tube. The samples were rinsed three times using TL-Hepes containing 0.01% polyvinyl alcohol (PVA). COCs were collected under a stereomicroscope and rinsed three times with *in vitro* maturation (IVM) medium (TCM-199 (Gibco) supplemented with 0.1% PVA, 3.05 mM D-glucose, 0.91 mM sodium pyruvate, 0.57 mM cysteine, 0.5 µg/ml Luteinizing Hormone (LH), 0.5 µg/ml Follicle-Stimulating Hormone (FSH), 10 ng/ml Epidermal Growth Factor (EGF) and 10 µg/ml gentamicin). COCs were then transferred to IVM medium covered with mineral oil and incubated at 38.5°C in an atmosphere of 5% CO_2_ in air. After 42–44 h of maturation culture, COCs were transferred to TL-Hepes containing 0.01% PVA and 0.1% hyaluronidase, vortexed to remove the cumulus cells. Oocytes with the first polar body were selected for further use.

### Oocyte Activation and Parthenogenetic Embryo Production

Oocytes were rinsed three times with activation medium (distilled water supplemented with 0.3 M mannitol, 1.0 mM CaCl_2_·2H_2_O, 0.1 mM MgCl_2_·6H_2_O, and 0.5 mM Hepes) and aligned within a chamber with two electrodes placed 0.5 mm apart, which was covered with activation medium. Two 30 µsec electrical pulses of 1.2 kV/cm were delivered. The activated oocytes were cultured in PZM-3 (108.0 mM NaCl, 10.0 mM KCl, 0.35 mM KH_2_PO_4_, 0.4 mM MgSO_4_·7H_2_O, 25.07 mM NaHCO_3_, 0.2 mM Na-pyruvate, 2.0 mM Ca(lactate)_2_·5H_2_O, 1.0 mM glutamine, 5.0 mM hypotaurine, 20 ml/L Eagle’s basal medium amino acid solution, 10 ml/L modified Eagle’s medium amino acid solution, 0.05 mg/ml gentamicin, 3 mg/ml BSA), covered with mineral oil, and cultured in 5% CO_2_ in air at 38.5°C. The parthenogenetic embryos were either transferred to recipient gilts after overnight culture for later fetus collection, or were cultured to the 8 to 16-cell stage. Eight to 16-cell stage embryos were used for blastomere karyotyping or cultured to blastocysts for stem-cell culture.

### Chromosome Spread Analysis of 8 to 16-cell Stage Blastomeres Derived from Porcine Parthenogenetic Embryos

The air-drying method for chromosome spread of mouse embryos [19] was used, with some modifications. Unlike porcine somatic cells and mouse embryos, chromosome spread of porcine blastomeres tended to be influenced by cytoplasmic components because porcine blastomeres are rich in lipid droplets. The 8-cell to 16-cell stages of parthenogenetic embryos were selected on the third day after activation and culture, and transferred to equilibrated PZM-3 supplemented with 20 ng/ml colchicine. After four hours of incubation, embryos were treated with Dulbecco's Phosphate Buffered Saline (DPBS) (Gibco) supplemented with 0.5% pronase to remove the zona pellucida. Individual blastomeres were dissected in DPBS containing 0.1% PVA. Blastomeres were incubated in hypotonic solution (distilled water supplemented with 0.075 M KCl, 0.1% PVA) at 37°C for 20 min, followed by treatment in pre-fixation solution (3∶1∶2, methanol:acetic acid:DPBS with 0.1% PVA) for 3 min. Pre-fixed blastomeres were transferred to fixation solution (3∶1, methanol: acetic acid) and incubated for 20 min. Fixed blastomeres were loaded onto slides under a stereomicroscope and three droplets of fixation solution were dropped onto each slide, followed by air-drying. Images of chromosome spread were captured using an inverted microscope.

### Parthenogenetic Embryonic Stem-like Cell Culture and Chromosome Spread Analysis

Parthenogenetic embryos were cultured in PZM-3 for 5–6 days. Good quality blastocysts were then selected for stem-cell culture. ICMs after trophectoderm cell removal, or entire PA blastocysts after zona pellucida removal, were cultured in porcine stem-cell medium I (α-MEM (Gibco) supplemented with 20% knockout serum replacement (KSR) (Gibco), 20 ng/ml rFGF, 40 ng/ml EGF, 1% 100× insulin-transferrin-selenium, 1% non-essential amino acid (NEAA) (Gibco), 50 µM 2-mercaptoethanol (Gibco) and 10 ng/ml activin A (Gibco)) or medium II (DMEM/F12 (Gibco) supplemented with 20% KSR, 20 ng/ml rFGF, 0.1 mM 2-mercaptoethanol and 1% penicillin-streptomycin solution (Gibco)). STO cells or mouse embryonic fibroblasts (MEFs) were used as feeder layers. The zona pellucida was removed as described above. The trophectoderm cells were removed according to Harris [20], using rabbit anti-porcine whole serum instead of rabbit anti-bovine whole serum. The ICMs or the whole blastocysts were seeded onto a mitotically inactivated feeder layer in 4-well dishes and incubated in 5% CO_2_ at 38.5°C. Approximately one week later, cells began to grow out from the ICMs or intact blastocysts. These cells were mechanically collected and examined microscopically [21]. The colonies were also digested enzymatically, but this method failed to maintain the cell population [22], [23]. The medium was changed every day, and cells were subcultured onto fresh feeder cells every 7 days.

The pluripotency of these stem-like cells was assayed by alkaline phosphatase (AP) staining and immunofluorescence staining for the pluripotency markers OCT4 and SSEA1. Importantly, for AP staining, the cells were fixed with 4% paraformaldehyde (PFA) for 2 min at room temperature (RT) in the dark. Fixed cells were washed three times with TBST (25 mM Tris–HCl, 0.14 M NaCl, 2.7 mM KCl, 0.1% Tween-20) and stained using the BCIP/NBT Color Development Substrate Kit (Promega) for 90 min at room temperature in the dark. Cells were washed with TBST to terminate the staining reaction. Stained cells were maintained in DPBS for observation. For immunofluorescence, the stem-like cells were briefly washed in DPBS and fixed in 4% PFA for 15 min at RT. After washing in DPBS, the cells were permeabilized in DPBS with 0.8% Triton X-100 at RT for 15 or 60 min. The cells were then incubated in DPBS with 10% goat serum for 1 h at RT to block nonspecific binding. This step was followed by overnight incubation with primary antibodies diluted in DPBS at concentrations of 1∶100 with 10% goat serum at 4°C. For SSEA-1 staining, the permeabilization step was omitted. The primary antibodies were rabbit anti-Oct-3/4 (Santa Cruz Biotechnology) and mouse anti-SSEA-1 (Millipore). The cells were then washed in DPBS and transferred to DPBS containing the appropriate Alexa Fluor-conjugated secondary antibodies (Invitrogen) at a dilution of 1∶300 and incubated at RT for 1 h. The cells were counterstained with 4′, 6-diamidino-2-phenylindole (DAPI). Negative controls used secondary antibodies alone. Laser-scanning microscopes (Nikon) were used for visualizing fluorescent signals.

Chromosome spread analysis of these stem-like cells was performed. The cells were cultured for 7 days after passage and the medium then replaced with fresh stem-cell culture medium containing 20 ng/ml colchicine. After 4 h, the cells were washed with DPBS, centrifuged and resuspended in 7 ml hypotonic solution in a conical tube and incubated for 35 min in a water bath. Freshly prepared fixation solution (1 ml) was added into the tube for pre-fixation at RT for 3 min, followed by three fixation steps, each for 20 min. The cell pellets were resuspended in fixation solution and one drop of fixation medium with cells was placed on each pre-cooled clean glass slide. Images of chromosome spread were captured using an inverted microscope.

### Parthenogenetic Embryo Transfer and Porcine Embryo Fibroblast Derivation

The PA embryos were cultured overnight and transferred into recipient gilts the following morning at the one-cell stage. The recipient gilt was either in standing estrus on the day of PA (day 1) or the day of embryo transfer (ET) (day 0). The mean number of PA embryos transferred was 170. The detailed surgical procedure has been previously reported [24]. The recipient gilts were sacrificed on day 30 of gestation. Fetuses were collected from the uteri of the gilts, rinsed three times with DPBS containing 1% penicillin-streptomycin solution, and the head and tail were removed. The main body of the fetus was placed in a 100 mm dish and minced into 1 to 2 mm pieces using sterilized scissors. The tissue was incubated in collagenase digestion medium (DMEM (Gibco) +200 µg/ml collagenase, 1% penicillin-streptomycin solution) in an incubator for 4–6 h. Finally, culture medium (DMEM, 10% fetal bovine serum (FBS) (Gibco), 1% penicillin-streptomycin solution) was added to terminate the digestion. The cells and medium were collected into a 15 ml conical tube and centrifuged for 5 min at 1,500 rpm. The supernatant was removed and the pellet was resuspended in culture medium and incubated in 5% CO_2_ in air at 38.5°C. After 24 h of culture, fibroblast cells were obtained by trypsin (Gibco) digestion and cryopreserved in medium containing FBS and 10% dimethylsulfoxide. The cell lines from different fetuses were stored in liquid nitrogen for further use.

### Sorting of Presumed Haploid Cells from Porcine Parthenogenetic Fibroblast Cell Lines

Cell lines were selected according to the preliminary results of the chromosome spread analysis of the porcine PA fetal fibroblasts. The frozen cells were thawed and cultured in a Petri dish. After one passage, 7–9×10^7^ cells were collected and sorted by flow cytometry (BD FACS ARIA II SORP) according to Elling’s method [11]. Briefly, cells were stained with 5 µg/ml Hoechst 33342, incubated at 38.5°C for 30 min followed by washing with DMEM and resuspension in a 15 ml centrifuge tube and sorted by fluorescence intensity. For each cell line, two groups of cells (P1 and P2) were collected and further cultured. Other cells were discarded.

### Chromosome Spread Analysis of Porcine Parthenogenetic Fetal Fibroblasts after Sorting

Colchicine was added (20 ng/ml) to the collected cell lines after 40–48 h of culture and the cell lines were incubated for a further 4 h. The chromosome spread analysis was conducted as described above.

### G-banding Chromosome Analysis of Blastomeres of Porcine Parthenogenetic 8- to 16-cell Embryos and Sorted Porcine Parthenogenetic Fetal Fibroblasts

Chromosome spread slides were placed into an oven at 60°C overnight. The chromosomes on the slides were digested in 0.0125% trypsin in DPBS (pH = 7.0) for 2 to 3 min, and then stained using Giemsa solution for 20 min. Images of the chromosome spread were captured using an inverted microscope using oil immersion lens at 1,000× total magnification.

### Microarray Analysis of Sorted Parthenogenetic Diploid Cell Lines

Total RNA from two sorted diploid cell lines was extracted using the TRIzol Reagent (Takara). The RNA of each cell line was aliquoted for two separate assays: microarray analysis and qPCR. Two wild-type female porcine fetal fibroblast cell lines served as controls. Each experimental cell line was analyzed in parallel with a control cell line. Two experimental cell lines and two control cell lines were used to perform a genomic expression analysis (microarray analysis) using the Affymetrix GeneChip Porcine Genome Array [25]. Three known porcine imprinted genes, *IGF2* (F:AAGAGTGCTCTTCCGTAG, R:TGTCATAGCGGAAGAACTTG), *PEG1* (F:TCTGAGCTGGAAAGAGTAGC, R:GGTGGACTTTGTGAGAGAG) and *PEG10* (F:GTTGTTAATGGCTGGAAGAG, R:AGTCACTTCCCCTTCCTAAG), were selected to validate the microarray results. Primers and RT-PCR reaction conditions were designed according to a previous study [26]. Beta actin (*ACTB*) served as the internal control (F:GTGGACATCAGGAAGGACCTCTA, R:ATGATCTTGATCTTCATGGTGCT).

Amplification and detection were carried out using the Roche Real-Time PCR system (Lightcycler 96) using a quantitative real-time PCR kit (Lightcycler PCR QC kit) under the following conditions: 95°C for 15 min, 40 cycles of denaturation at 95°C for 15 s, annealing at 60°C for 30 s, and extension at 72°C for 30 s. The PCR reaction mixture (10 µl) consisted of 100 pmol of forward and reverse primers and 1 µl of cDNA (reverse transcribed from total RNA of sorted cell lines) [26]. Samples were analyzed in triplicate. All the threshold cycle values of imprinted genes were normalized to that of the *ACTB* gene, and relative expression ratios were calculated via the 2^−ΔΔCt^ method.

### Statistical Analysis

The attachment and colony formation rate and the percentage of different cell type were compared using the chi-square test. The quantitative real-time PCR results of different genes in different cell lines were compared using the *t*-test.

## Results

### Chromosome Spread and G-banding of Blastomeres from 8-cell to 16-cell PA Embryos

**Figure 1:**
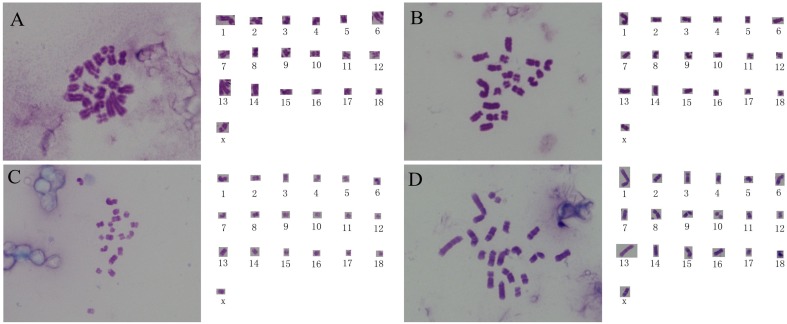
The chromosome spread and G-banding analyses of blastomeres from 8-cell to 16-cell parthenogenetically activated embryos. (A and B) The chromosome spread and corresponding chromosome alignment of haploid blastomeres; (C and D) The G-banding karyotype and corresponding chromosome alignment of haploid blastomeres.

We obtained chromosome spread from 81 blastomeres and 48.15% (39/81) of the blastomeres exhibited a haploid karyotype (Fig. 1), 8.64% (7/81) exhibited a diploid karyotype (Fig. S1A, B), and 1.23% (1/81) blastomeres were tetraploid (Fig. S1C). The remaining blastomeres (34/81) were aneuploid (Fig. S1D–S1I). All the observed haploid cells contained 19 chromosomes, without loss or gain of any chromosome (Fig. 1A, B). Chromosome spread are not enough to discern the integrity all of the chromosomes, so to validate the genome integraty of the observed haploid cells, G-banding analysis was performed. G-banding analysis confirmed that the haploid blastomeres did contain intact haploid genomes (Fig. 1C, D) [27], [28]. These results verified that haploid cells with intact genomes existed in the early-stage PA embryos. This initial analysis also suggested that authentic haploid cells could be obtained by culturing the PA embryos containing haploid cells to generate haploid stem-like cells, or by transferring the haploid cell contained PA embryos into recipient gilts and collecting fetuses after 30 days to obtain haploid fetal fibroblasts.

### Embryonic Stem-like Cells Derived from PA Embryos and Chromosome Spread

**Figure 2:**
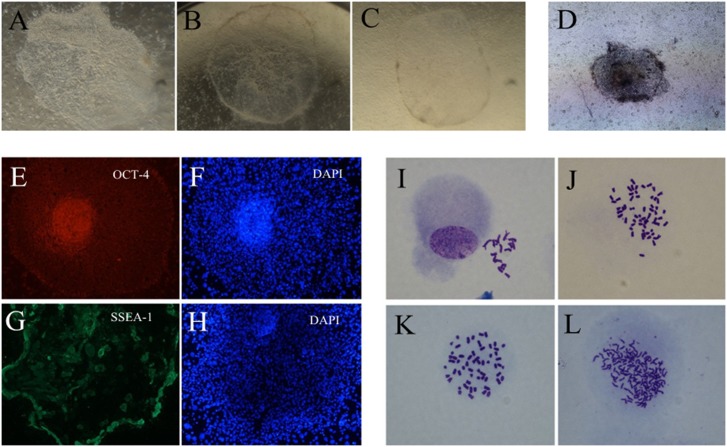
The morphology, immunofluorescence staining and chromosome spread of PA embryo-derived embryonic stem-like cells. (A–C) The primary stem-like cell colonies: A from whole blastocyst, B and C from inner cell masses (ICMs). The stem-like cell colony from whole blastocysts contains large amounts of lipid droplets. (D) A stem-like cell colony that is alkaline phosphatase positive. (E and F) OCT4 immunofluorescence staining and corresponding DAPI staining. (G and H) SSEA-1 immunofluorescence staining and corresponding DAPI staining. (I–L) The aneuploid chromosome spread of the ICM-derived cell lines, contain 17, 57, 54 and uncounted chromosomes, respectively.

Both α-MEM and DMEM culture media supported the attachment and outgrowth of whole blastocysts or ICMs. However, the whole blastocysts had a higher colony formation rate when STO cells were used as feeder cells, compared to the use of MEFs. ICMs had higher attachment rates with α-MEM as medium when STO cells were used as feeder cells, compared to MEF feeder cells. The whole blastocysts had higher attachment rates and outgrowth rates than the ICMs when the same medium was used. However, ICMs could not attach to MEFs when DMEM was used (Table 1). Whole blastocyst-derived stem-like cell colonies contained large amounts of lipid droplets (Fig. 2A) compared to ICM-derived colonies (Fig. 2B, C). A total of six independent ES-like cell populations were obtained and chromosome spread analysis was performed. Four of the populations were derived from ICMs cultured in α-MEM with STO feeder cells and two populations were derived from whole blastocysts cultured in DMEM with STO feeder cells; these latter two cell lines were cultured for more than 20 passages. The cell lines were positive for AP activity (Fig. 2D), and stained positive for pluripotency markers such as OCT-4 (POU5F1) and SSEA-1 (Fig. 2E–2H). Many abnormal karyotypes were detected among the six cell lines (Fig. 2I–2L). No chromosome spread with exactly 19 chromosomes was found in any of the cell lines. Four ICM-derived cell lines (pSLC3, 4, 5 and 6) contained high percentages of cells with more than 38 chromosomes. The overall percentage of aneuploid cells with >38 chromosomes in the four ICM-derived cell lines was 50.94%, the percentage of diploid cells was 24.53%, and aneuploid cells with chromosome numbers between haploid and diploid was 20.75%. Aneuploid cells with a chromosome number less than haploid accounted for only 3.77% of the population (Table 2). In the two whole blastocyst-derived cell lines, the overall percentage of aneuploid cells with >38 chromosomes was 82.76%, diploid cells was 0%, and aneuploid cells with chromosome numbers between haploid and diploid was 6.90%. Aneuploid cells with a chromosome number less than haploid were 10.34%. Regarding the individual cell line, the majority of the pSLC1 cell line was diploid. The pSLC2 cell line had a high percentage of cells with chromosome numbers falling between the normal haploid (19) and diploid (38) chromosome number. In the pSLC6 cell line, 56.52% (13/23) of the cells had exactly 76 chromosomes (tetraploid) (Table 2); this cell line may actually be a trophoblast cell line. Taken together, cells derived from PA oocytes tended to develop polyploid and aneuploid cells. The results confirmed that PA-derived cells cultured in current porcine ES cell culture media had high degrees of genome instability.

**Table 1:** The attachment and outgrowth rate of porcine whole blastocysts or inner cell masses (ICMs) in different media with different feeder cells.

	DMEM						αMEM		
	Whole blastocyst			ICM			ICM		
	Blastocysts implanted	Attachment rate	Colonies formed	Blastocysts implanted	Attachment rate	Colonies formed	Blastocysts implanted	Attachment rate	Colonies formed
MEF	117	28 (23.9%)	2 (1.7%)^a^	20	0 (0)	0 (0)	30	2 (6.7%)^a^	2 (6.7%)
STO	149	52 (34.9%)^d^	20 (13.4%)^b,d^	30	5 (16.7%)^c^	2 (6.7%)^c^	30	9 (30.0%)^b^	5 (16.7%)

a,b Different superscripts within a column are significantly different: a<b, P<0.05.

c,d Different superscripts within a row are significantly different: c<d, P<0.05.

**Table 2:** Chromosome spread of stem-like cell lines derived from ICMs and whole blastocysts.

Cell lines	Aneuploid (chromosome number<19)	Haploid (chromosome number = 19)	Aneuploid(19<chromosome number<38)	Diploid (chromosome number = 38)	Aneuploid (chromosome number>38)	Total
pSLC1	1	0	1	11	2	15
pSLC2	0	0	6	0	1	7
pSLC3	1	0	0	0	11	12
pSLC4	0	0	4	2	13	19
subtotal	2 (3.77%)^a^	0 (0)^a^	11 (20.75%)^a,b^	13 (24.53%)^a,b^	27 (50.94%)^b^	53
pSLC5	1	0	2	0	3	6
pSLC6	2	0	0	0	21*	23
subtotal	3 (10.34%)^a^	0 (0)^a^	2 (6.90%)^a^	0 (0)^a^	24 (82.76%)^b^	29
total	6 (7.32%)^a,b^	0 (0)^a^	12 (14.63%)	13 (15.85%)	51 (61.20%)^b^	82

a,b Different superscripts within a row are significantly different: a<b, P<0.05.

^*^Among these 21 cells observed, 13 with exactly 76 chromosomes.

The pSLC1–pSLC4 cell line is derived from inner cell masses. The pSLC5 and pSLC6 cell lines are derived from whole blastocysts.

### PA Fetus Collection and Fibroblast Chromosome Spread

**Figure 3:**
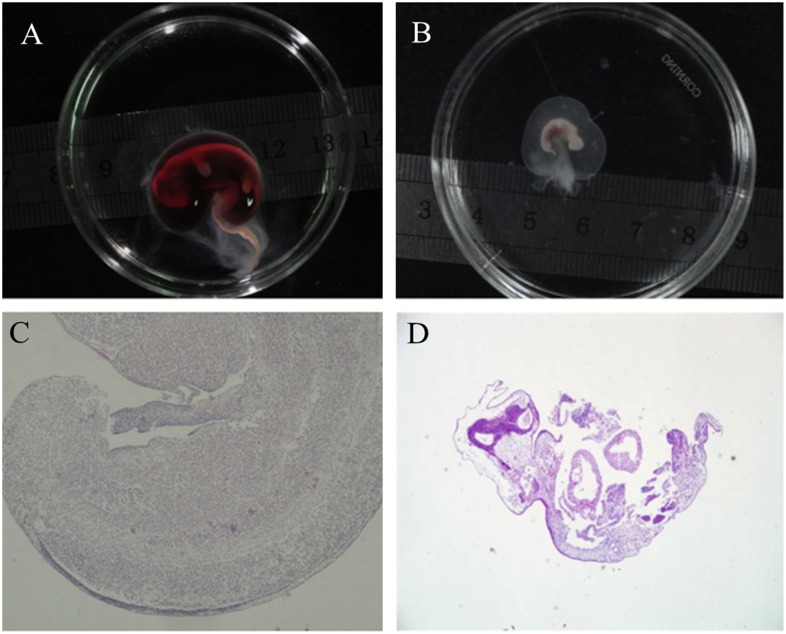
The PA fetus morphology and HE staining. (A) A large PA fetus with hemolysis. (B) A retarded ultra-small PA fetus. (C and D) HE staining of paraffin-embedded sections of large and ultra-small PA fetuses. The cell apoptosis is shown in the ultra-small fetus.

PA embryos were transferred to oviducts of four gilts (195, 128, 165 and 197 embryos transferred). Three of the gilts developed pregnancies and a total of 50 fetuses were collected on day 30. The body size of the fetuses ranged from 2–24 mm (Table S1) (Fig. 3A, B). Some fetuses were undergoing hemolysis (Fig. 3A). The largest 30 day parthenogenetic fetus possessed a body size (24 mm) similar to that of a normal 19 day fertilized fetus (20±5 mm), which was reported by Kure-Bayashi. [29]. We divided fetuses into two groups according to body size: 15–24 mm (32.00%, 16/50), and fetuses smaller than 15 mm (68.00%, 34/50) (Table 3). Nine fetuses of various sizes were fixed for paraffin sections and HE staining. The parthenogenetic fetus (body size 24 mm) had a morphology (Fig. 3C) similar to the naturally fertilized fetuses and smaller fetuses exhibited cell apoptosis (Fig. 3D). Another 41 fetuses were used for fibroblast culture. Fibroblast cell lines were harvested from 37 fetuses. The karyotypes of the fetal fibroblasts were variable: chromosome number varied from 11 to 76. Aneuploid cells with chromosome number between 19 and 38 were the main cell type (59.48–60.91%) (Fig. S2A–S2F), and the diploid cells were the second cell type (16.17%–22.73%) (Fig. S2G–S2I) (Table S2), which differed from that observed within the parthenogenetic stem-like cells. A low percentage of cells with 19 chromosomes were detected in 18.52% of the fetus-derived cell lines and the percentage varied from 3.45% to 8.33%. The fetal fibroblasts isolated from fetuses of gilt 3 contained a higher percentage of cells with 19 chromosomes compared with other gilt-derived cell lines (Table S2), and were therefore used for the following experiments.

**Table 3:** The body size of day 30 porcine parthenogenetic fetuses derived from transfer of PA embryos to three gilts.

	Fetus (size<15 mm)	Fetus (size>15 mm)	Total
rP-1 (recipient gilt 1)	17 (100%)^b^	0 (0)^a^	17
rP-2 (recipient gilt 2)	11 (57.89%)^a^	8 (42.11%)^b^	19
rP-3 (recipient gilt 3)	6 (42.86%)^a^	8 (57.14%)^b^	14
Total	34 (68.00%)	16 (32.00%)	50

a,b Different superscripts within a column are significantly different, a<b; P<0.05.

### Haploid Fibroblast Enrichment and Chromosome Spread

**Figure 4:**
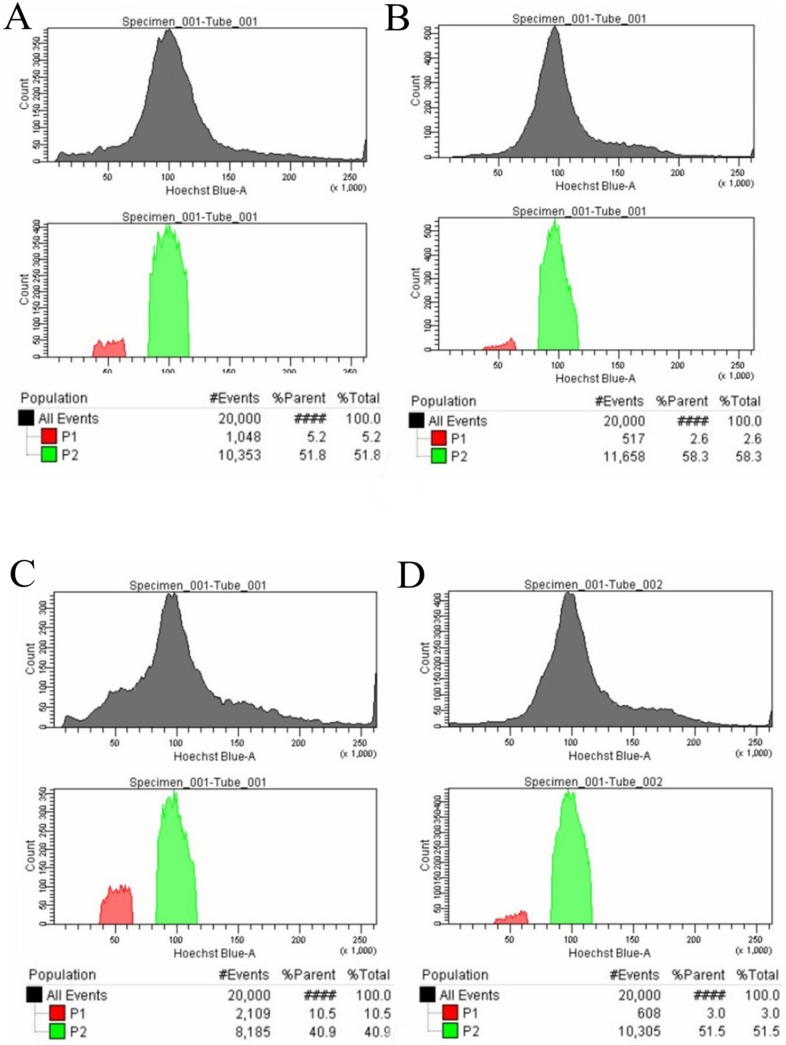
The flow cytometry sorting results of PA embryo-derived fetal fibroblasts. (A and B) The sorting of cell line pPEF3-2 and the corresponding control fPEF5. (C and D) The sorting of cell line pPEF3-5 and its corresponding control fPEF6. The results show that PA cell lines indeed contain more cells with decreased (less than diploid) DNA content.

Two cell lines from gilt 3-derived fetuses, pPEF3-2 and pPEF3-5, were used in these experiments. These cell lines were sorted using Elling’s method [11] to obtain a haploid cell enriched population and a diploid cell enriched population. The four cell populations obtained were named pPEF3-2 1N, pPEF3-2 2N, pPEF3-5 1N and pPEF3-5 2N (Fig. 4A–4D). These cell lines were cultured for 40–48 h after sorting to eliminate the effects of the sorting procedure on both chromosome spread capture and the global gene expression profile of the cell lines. Two wild-type female porcine fetal fibroblast (fPEF) cell lines (fPEF 5 and fPEF 6) served as controls. The cells were subjected to chromosome spread and microarray analyses. The results indicated that the pPEF3-2 1N cell line contained 5.97% (4/67) haploid cells and 34.33% (23/67) diploid cells, whereas the pPEF3-2 2N line had no haploid cells and 89.06% (57/64) diploid cells. The pPEF3-5 1N line contained 5.63% (4/71) haploid and 35.21% (25/71) diploid cells, whereas the pPEF3-5 2N line had no haploid and 86.36% (57/66) diploid cells (Table 4). These results showed that haploid fibroblasts had not been enriched and the diploid fibroblasts were enriched efficiently.

**Table 4:** The karyotype distribution of porcine parthenogenetic fetal fibroblasts after sorting.

Cell lines	Aneuploid (chromosome number<19)	Haploid(chromosome number = 19)	Aneuploid(19<chromosome number<38)	Diploid(chromosome number = 38)	Aneuploid (chromosome number>38)	Total
pPEF3-2 1N	5 (7.46%)^a,c^	4 (5.97%)^c^	29 (43.28%)^a,d^	23 (34.33%)^a,d^	6 (8.96%)^c^	67
pPEF3-2 2N	2 (3.13%)^a,c^	0 (0)^c^	5 (7.81%)^a,c^	57 (89.06%)^b,d^	0 (0)^c^	64
pPEF3-5 1N	19 (26.76%)^b,d^	4 (5.63%)^c^	22 (30.99%)^b,d^	25 (35.21%)^a,d^	1 (1.41%)^c^	71
pPEF3-5 2N	2 (3.03%)^a,c^	0 (0)^c^	6 (9.09%)^a,c^	57 (86.36%)^b,d^	1 (1.52%)^c^	66

a,b Different superscripts within a column are significantly different: a<b, P<0.05.

c,d Different superscripts within a row are significantly different: c<d, P<0.05.

**Figure 5:**
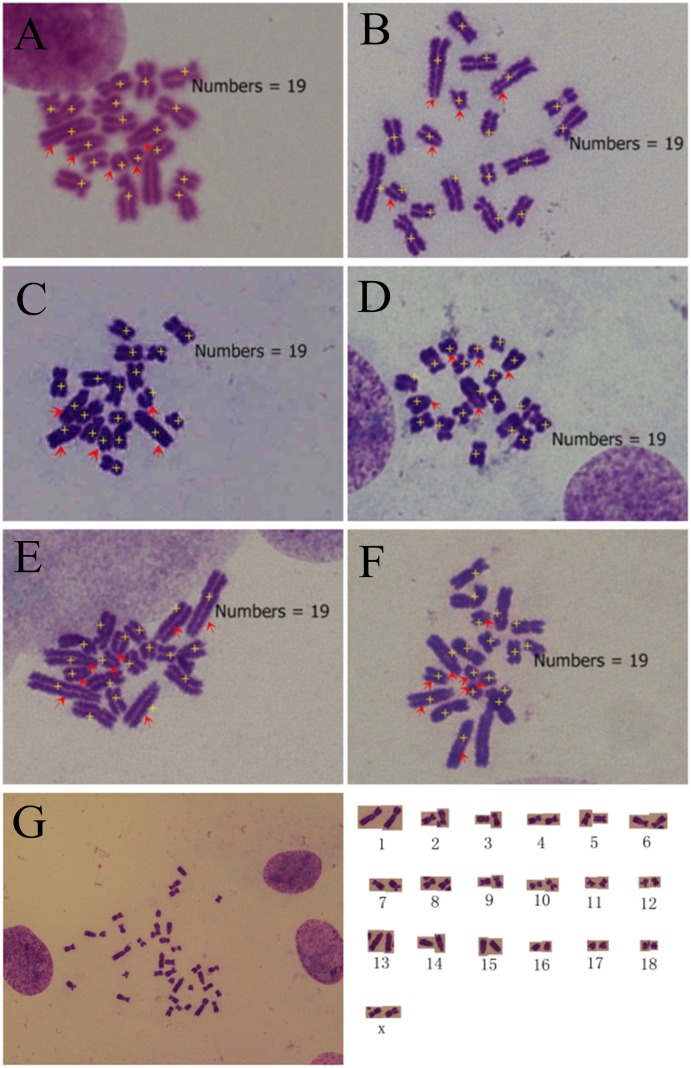
The chromosome spread of PA embryo-derived fibroblasts. (A–D) Haploid PA cells with an abnormal chromosome spread lack one telocentric chromosome. (E and F) The haploid cells with other kinds of abnormal chromosome spread. The red arrow indicates a telocentric chromosome. (G) The diploid cell with normal karyotype.

Six perfect haploid chromosome spread were analyzed for genome integrity, one from the pPEF3-1 line before sorting, three from the pPEF3-2 1N line, and two from the pPEF3-5 1N line after sorting. Among the haploid fibroblasts, 66.67% (4/6) of these cells contained 19 chromosomes including five telocentric chromosomes (Fig. 5A–5D). The other two haploid chromosome spread had more than 6 telocentric chromosomes (Fig. 5E, F). Normally, the intact haploid porcine genome should contain six telocentric chromosomes [28]. The diploid chromosome spread from pPEF3-2 2N and pPEF3-5 2N cell lines showed a normal diploid karyotype (Table 4) (Fig. 5G). These two cell lines had similar percentage of normal diploid fibroblasts (89.06% and 86.36%) compared with wild type of porcine fibroblast cell lines, which contain nearly 90% of the cells with normal diploid karyotype; by comparison, the pPEF3-2 2N and pPEF3-5 2N lines were considered normal diploid cell lines. The pPEF3-2 1N and pPEF3-5 1N cell lines had high percentages of abnormal karyotypes and a very low percentage of haploid and diploid karyotypes.

### G-banding Analysis

**Figure 6:**
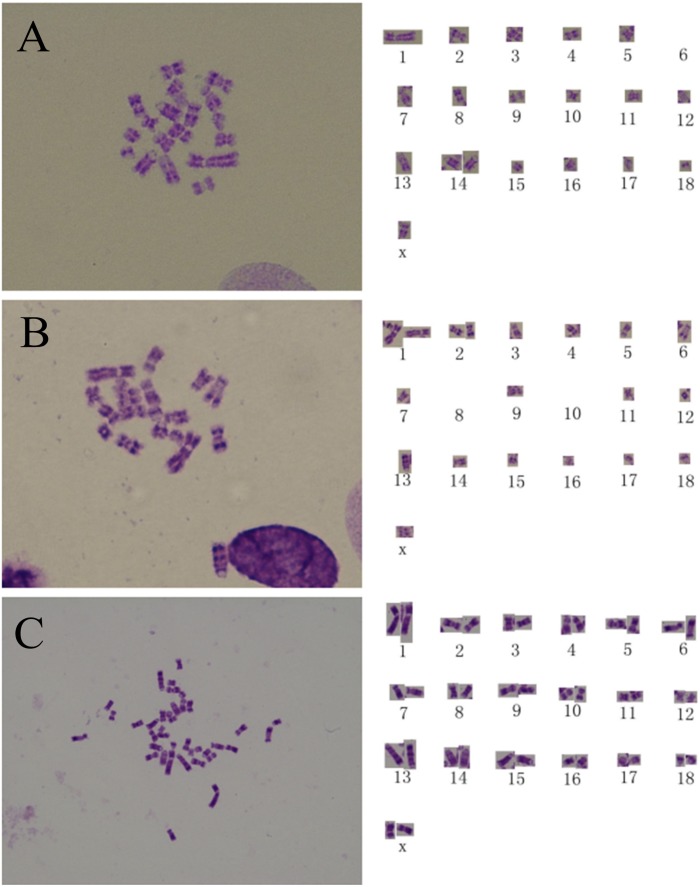
The G-banding analysis of PA embryo-derived fibroblasts. (A) The haploid cell lacks chromosome 6, and has gained an extra chromosome 14. (B) The haploid cell lacks chromosome 8 and 10, and has gained an extra chromosome 1 and 2. (C) G-banding of a diploid cell derived from sorting indeed showed a normal karyotype.

Since most of the sorted haploid cells lacked one telocentric chromosome compared to the normal porcine haploid genome, we performed a low resolution G-banding analysis and chromosome alignment on three unsorted primary fibroblast cell lines to determine whether some haploid cells possessed intact genomes or exhibited consistent loss of particular chromosomes. A total of 386 clear G-band images were captured. The overall percentage of haploid cells was 2.33% (9/386). None of the haploid cells exhibited genomic integrity because the cells showed losses or gains of chromosomes. For example, in one cell, chromosome 6 was absent and an extra chromosome 14 was present (Fig. 6A), and in another cell, chromosomes 8 and 10 were absent and an extra chromosome 1 and 2 were present (Fig. 6B). The G-banding of sorted diploid cells showed a normal diploid karyotype (Fig. 6C).

### Transcription Profile of the Sorted Cell Lines

**Figure 7:**
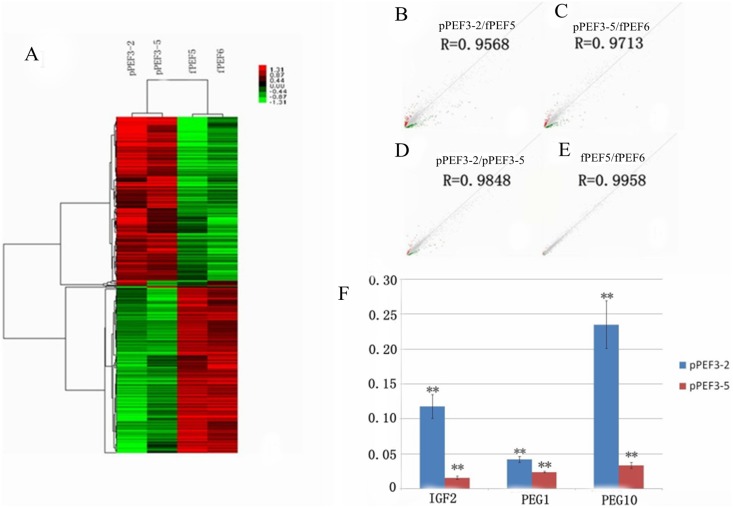
The microarray and RT-PCR analysis of PA embryo-derived fetal fibroblasts. (A) The heat map of PA embryo-derived fetal fibroblast cell lines and the corresponding female control cell lines. (B–E) The dot-blot comparison of different cell lines. (F) The real-time PCR results of imprinted genes. The fold change of three genes, the y-axis represents fold change.

Gene expression heat maps and gene clusters were generated using a 2-fold change in gene expression relative to control cell lines (Fig. 7A). The pPEF3-2 2N cell line had 1,242 genes with >2-fold change in expression compared to the control cell line fPEF5. The pPEF3-5 2N cell line had 1,083 genes with >2-fold change in expression compared to the control cell line fPEF6. The pPEF3-2 2N and pPEF3-5 2N cell lines had 338 genes with a 2-fold change in common: 148 genes were downregulated and 190 were upregulated. The relevance plot results showed that the two control fPEF cell lines have a stable and similar expression profile. The PA fetus cell lines had similar transcriptional profiles that were distinctly different from the control cell lines (Fig. 7B–7E).

To further validate the microarray results and to investigate whether the parthenogenetic diploid fibroblast cell lines are suitable for the purpose of discovering unknown paternally imprinted genes, we performed qPCR. We selected three known paternally imprinted genes (*IGF2*, *PEG1* and *PEG10*) and examined their expression in the two parthenogenetic diploid cell lines. All three genes had significantly lower expression in the PA fibroblast cell lines compared to the control cell lines (Fig. 7F). *ACTB* was used as an endogenous control.

The most downregulated genes in the overlapping subset of genes of the two parthenogenetic diploid cell lines are listed in Table 5 and include two known porcine paternally imprinted genes (*PEG10*, *SGCE*) that were downregulated significantly. Additional genes might be selected as candidate paternally imprinted genes for further investigation (Table 5).

**Table 5:** Ten most downregulated genes in porcine parthenogenetic diploid fetal fibroblasts.

	Probe set ID	Gene name	Gene title	Gene expression fold change	
				pPEF3-2 2N	pPEF3-5 2N
1	Ssc.13476.1.A1_at	*PEG10*	Paternally expressed 10	0.0969	0.0232
2	Ssc.3772.1.A1_at	*SGCE*	Sarcoglycan, epsilon	0.1591	0.0613
3	Ssc.6974.1.A1_at	*SYTL2*	Synaptotagmin-like 2	0.1552	0.1334
4	Ssc.9291.1.A1_at	*PALMD*	Palmdelphin	0.2922	0.1725
5	Ssc.1534.1.A1_at	*SORBS1*	Sorbin and SH3 domain containing 1	0.0805	0.1838
6	Ssc.18021.1.A1_at	*PSAP*	Prosaposin	0.3341	0.2164
7	Ssc.27593.1.S1_at	*TGFB3*	Transforming growth factor, beta 3	0.1047	0.2187
8	Ssc.23247.1.S1_at	*MYLK*	Myosin light chain kinase	0.1468	0.2204
9	SscAffx.32.1.S1_at	*ND4L*	NADH dehydrogenase subunit 4L	0.2865	0.2800
10	Ssc.4118.2.S1_at	*MIR145*	MicroRNA mir-145	0.1808	0.2930

## Discussion

We successfully characterized chromosome spread of blastomeres of porcine parthenogenetic embryos, parthenogenetic embryo-derived ES-like cells, and parthenogenetic embryo fibroblasts. We confirmed that haploid cells with an intact haploid genome only exist in preimplantation parthenogenetic embryos; prolonged culture, either *in vivo* or *in vitro* that resulted in diploid, polyploid or aneuploid cells.

At the early cleavage stage, porcine parthenogenetic embryos maintained 48.15% haploid blastomeres, and all the haploid cells were authentic haploidy. The real ratio of haploid cells is potentially higher than observed because chromosomes tended to be lost when the spread were made. Therefore, the haploid karyotype of parthenogenetic embryos during the first several cleavages is much more stable than parthenogenetic embryo-derived cells after prolonged culture. The haploid blastomeres could be used for preimplantation genetic diagnosis to identify the occurrence of genetic diseases. Some blastomeres derived from one particular oocyte could be used for preimplantation genetic diagnosis, and the others used to reconstruct new oocytes [30], if the diagnosed blastomeres are without high-risk genetic disease; thus the risk of transmitting a genetic disease to the offspring could be reduced. These haploid blastomeres could be candidate materials to produce semi-cloned animals to take advantage of the reproductive ability of an excellent dam. The haploid cells could also be used for forward and reverse genetic screens, because any recessive mutation of an essential gene will show a clear phenotype because a second allele is absent.

Porcine parthenogenetic stem-like cells exhibited a wide variety of abnormal karyotypes and no cells with the exact haploid complement of 19 chromosomes were detected. Among four ICM-derived cell lines, only 24.53% (13/53) cells possessed a diploid chromosome spread. None of diploid cell was detected in two whole blastocyst-derived cell lines. These characterizations of porcine parthenogenetic stem cell lines are significantly different from that of trophectoderm stem cells [31] and mouse haploid stem cells [11]. Improper stem-cell culture conditions could be an important factor inducing these chromosome abnormalities, but might be not the only reason [32].

Attempts to derive mouse haploid cell lines began in 1974 [33]. Kaufman found that haploid cells exist at an early stage of embryogenesis, but cell lines derived from single pronucleus embryos showed a diploid karyotype [34]. Therefore, the prevailing notion at that time was that normal mammalian haploid cells could not survive in the suboptimal stem-cell culture medium. Breakthroughs by three groups of researchers in recent years have demonstrated that mouse haploid cells can be maintained *in vitro*. The 2i stem-cell culture medium developed by Ying in 2008 fostered the development of mouse ground state embryonic stem cells [35]. Leeb derived haploid embryonic stem cells from mouse parthenogenetic embryo using the 2i medium [10] and Elling derived haploid embryonic stem cells from parthenogenetic embryo with leukemia inhibitory factor (LIF) culture system [11]. Li et al. derived an androgenetic haploid embryo stem-cell line using 2i medium and performed chromosome G-banding experiments to validate the genome integrity of the cell lines [9], [12]. These successes in generating mouse haploid cells depended on improvements in mouse stem-cell culture conditions. Due to a poor understanding of the pathways and mechanisms mediating porcine embryonic stem-cell generation and the current suboptimal porcine stem-cell culture conditions, porcine germline competent embryonic stem cells have not been generated [36]. Fujishiro derived naïve-like porcine-induced pluripotent stem-cell lines [37], but this stem-cell line is not a ground state stem-cell line; thus breakthroughs in the development of porcine haploid stem-cell lines will be preceded by the development of a porcine naïve stem-cell culture system [37], [38].

Since haploid porcine ES-like cells were not successfully generated, we transferred porcine parthenogenetic embryos into surrogate mothers, later collected the fetuses, isolated and cultured fibroblasts. Cell sorting was then used to obtain a larger population of porcine haploid cells than that was available from the embryo blastomeres [39]. The karyotypes of the fetal fibroblasts showed different abnormalities, compared with stem-like cells. A low percentage of cells with 19 chromosomes were detected and, unfortunately, these cells did not contain an intact porcine haploid genome. The G-banding experiments confirmed the random character of chromosome loss and gain.

Further culture of the porcine haploid cells resulted in an extremely unstable cell karyotype. Chromosome instability in PA oocytes occurred at the cleavage and embryogenesis stages [40]. The instability of PA oocyte-derived diploid cells reportedly results from the lack of a centriole which is inherited through the sperm in mammalian species, with the exception of rodents [41]. Whether the failure to derive a porcine haploid cell line was caused by the lack of a male centriole within the haploid cell warrants further investigation. Recently, monkey haploid stem-cell lines were established by Yang. These cell lines could be maintained without sequential sorting for more than 100 days [13], which is quite different from the mouse. The derivation and maintenance of haploid cells has been highly variable among different species.

PA oocytes implanted into recipient gilts were shown to develop for up to 30 days in pigs [29]. The day 30 fetuses we derived from the presumed haploid PA oocytes had a significantly smaller body size than the fetuses from diploid parthenogenetic embryos [29], but despite this smaller size, almost every fetus produced numerous fibroblasts. The PA fetus-derived fibroblasts had higher percentage of diploid cells compared with PA embryo-derived blastomeres. The majority of diploid fibroblasts within the chimeric fetus are probably derived from auto-diploidization of haploid cells [11] and the lack of paternally imprinted genes could be the major cause of the lagging development of PA fetus body size [42]. Within each litter of fetuses, we found that almost half of them had a significantly smaller size.

Here, we identified eight genes with significant down regulation that could be candidate paternally imprinted genes. The microarray analysis combined with qPCR results confirmed that the diploid parthenogenetic fibroblasts might be suitable for predicting new paternally imprinted genes. Identification of imprinted genes by SNPs is widely used. Few porcine imprinted genes have been identified due to the lack of both optimal materials and known SNPs information, compared to human and mouse data. The porcine parthenogenetic diploid fibroblasts obtained in this study might be an alternative source for imprinted gene research.

## Supporting Information

Figure S1 **The chromosome spread of blastomeres from 8-cell to 16-cell parthenogenetically activated embryos.** (A and B) The chromosome spread of diploid blastomeres. (C) The chromosome spread of tetraploid blastomere. (D–I) The chromosome spread of neuploid blastomeres, contain 9, 23, 36, 48, 62 and uncounted chromosomes, respectively.
(TIF)

Figure S2 **The chromosome spread of PA embryo-derived fibroblasts.** (A–F) The chromosome spread of aneuploid blastomeres, contain 15,16,17,22,22 and 28 chromosomes, respectively. (G–I) The chromosome spread of diploid blastomeres, respectively.
(TIF)

Table S1 **The detailed information of porcine parthenogenetic fetuses.**
(XLS)

Table S2 **The karyotypes of porcine parthenogenetic fetal fibroblasts before sorting.**
(XLS)
