# Supplementary figures and images for: Karyotype Characterization of In Vivo- and In Vitro-Derived Porcine Parthenogenetic Cell Lines
Source: PLoS One. 2014 May 20;9(5):e97974. doi: 10.1371/journal.pone.0097974 (PMC4028241; doi:10.1371/journal.pone.0097974)

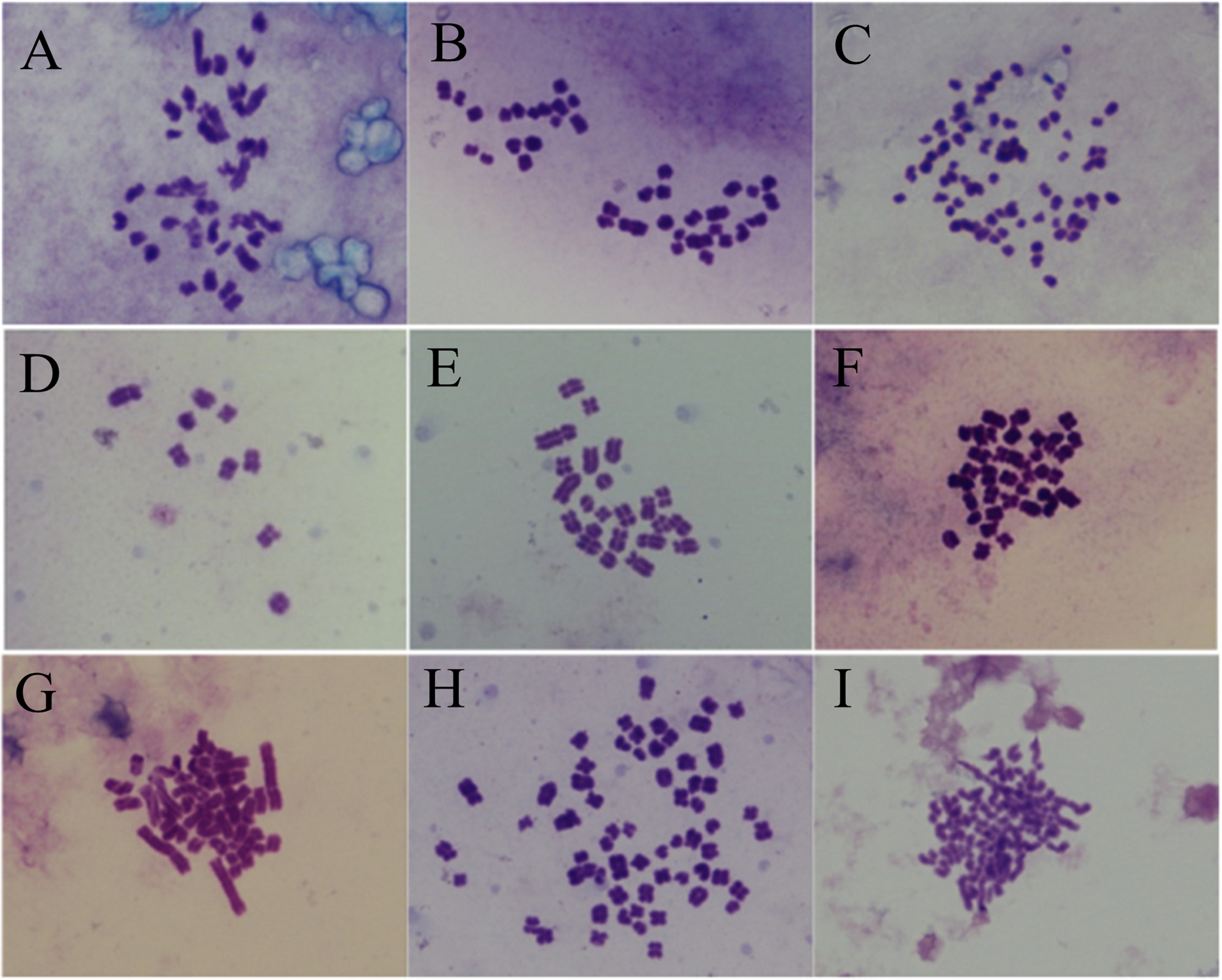

Supplement: Figure S1 — The chromosome spread of blastomeres from 8-cell to 16-cell parthenogenetically activated embryos. (A and B) The chromosome spread of diploid blastomeres. (C) The chromosome spread of tetraploid blastomere. (D–I) The chromosome spread of neuploid blastomeres, contain 9, 23, 36, 48, 62 and uncounted chromosomes, respectively. (TIF) [file pone.0097974.s001.tif]

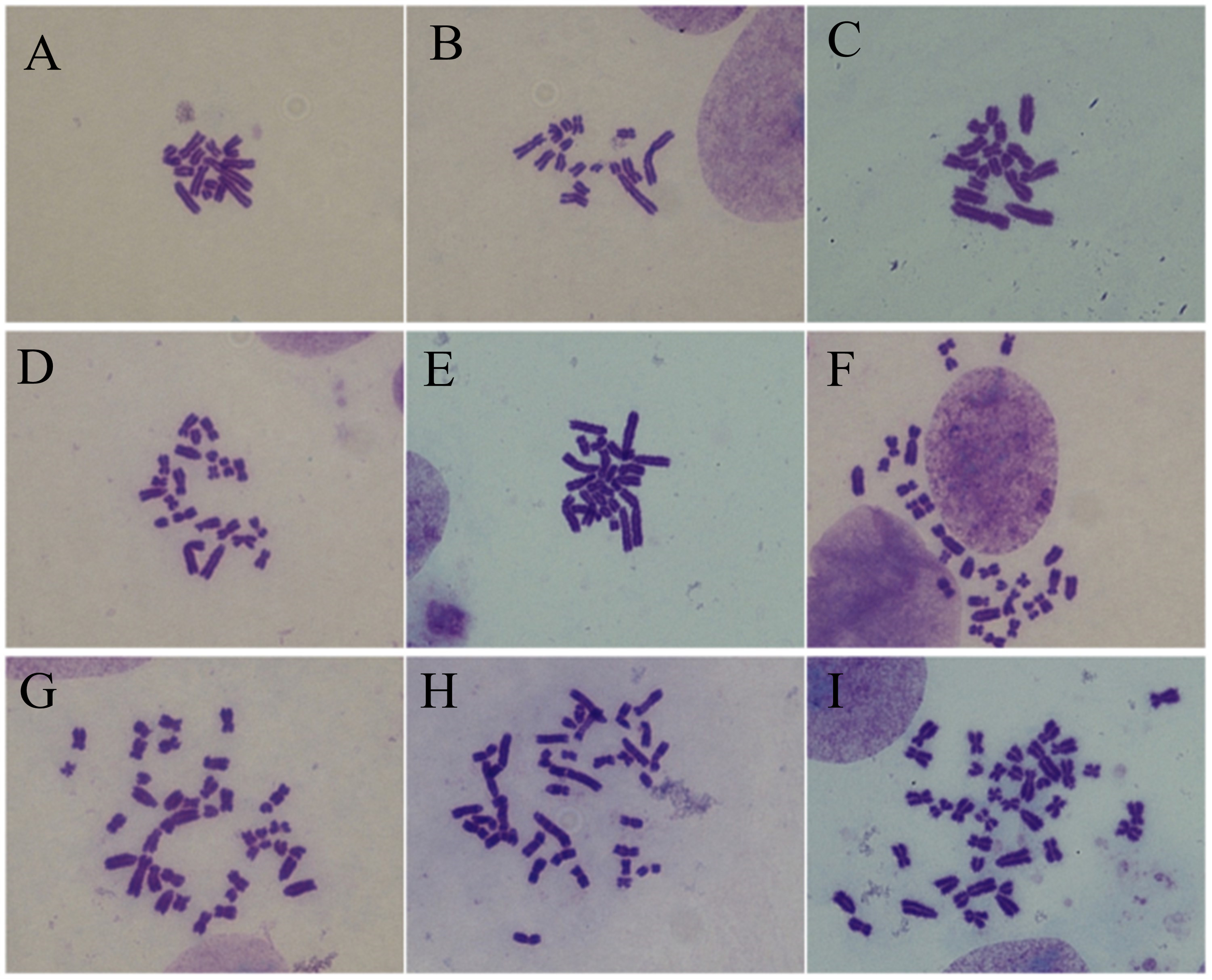

Supplement: Figure S2 — The chromosome spread of PA embryo-derived fibroblasts. (A–F) The chromosome spread of aneuploid blastomeres, contain 15,16,17,22,22 and 28 chromosomes, respectively. (G–I) The chromosome spread of diploid blastomeres, respectively. (TIF) [file pone.0097974.s002.tif]
